# Individualized Fortification Influences the Osmolality of Human Milk

**DOI:** 10.3389/fped.2018.00322

**Published:** 2018-10-31

**Authors:** Nathalie Kreins, Rachel Buffin, Diane Michel-Molnar, Veronique Chambon, Pierre Pradat, Jean-Charles Picaud

**Affiliations:** ^1^Neonatal Intensive Care Unit, Croix Rousse University Hospital, Hospices Civils de Lyon, Lyon, France; ^2^Regional Human Milk Bank, Croix Rousse University Hospital, Hospices Civils de Lyon, Lyon, France; ^3^Centre de Biologie, Hôpital de la Croix Rousse, Hospices Civils de Lyon, Lyon, France; ^4^Center for Clinical Research, Croix Rousse University Hospital, Hospices Civils de Lyon, Lyon, France; ^5^CarMeN Unit, Inserm U1060, INRA U1397, Claude Bernard University Lyon 1, Pierre Bénite, France; ^6^Faculté de Médecine Lyon Sud Charles Merieux, Université Claude Bernard Lyon 1, Pierre Bénite, France

**Keywords:** fortifier, growth, nutrition, prematurity, protein, necrotizing enterocolitis, breastmilk, energy

## Abstract

**Background:** Fortification of human milk (HM) increases its osmolality, which is associated with an increased risk of necrotizing enterocolitis. The impact of new fortifiers on osmolality is not well-known, nor are the kinetics regarding the increase in osmolality.

**Aim:** To determine the optimum fortifier composition for HM fortification by measuring the osmolality of fortified HM made with three powder multicomponent fortifiers (MCFs) and a protein fortifier (PF).

**Methods:** The osmolality of HM was assessed at 2 (H2) and 24 (H24) h after fortification to compare the effects of MCF (MCF1–3) and PF used in quantities that ensured that infants' nutrient needs would be met (MCF: 4 g/100 ml HM; PF: 0.5 g or 1 g/100 ml HM). To evaluate the early kinetics associated with the osmolality increase, the osmolality of HM fortified with MCF1 or MCF2 was also measured at 0, 1, 5, 10, 15, 20, 30, 40, 50, 60, 90, and 120 min after fortification.

**Results:** The osmolality increased significantly immediately after fortification, depending on the type of fortification used and the quantity of MCF and PF used, rather than the time elapsed after fortification. The maximum value at H24 was 484 mOsm/kg. The mean increase in osmolality between H2 and H24 was 3.1% (*p* < 0.01) (range: 0.2–10.8%). Most of the increase (>70%) occurred immediately after fortification.

**Conclusion:** When choosing a fortifier, its effect on HM osmolality should be considered. As most of the increase in osmolality occurred immediately, bedside fortification is not useful to prevent the increase in osmolality, and further research should focus on improving fortifier composition.

## Introduction

Human milk (HM) is the gold standard for premature infants' nutrition during hospitalization, but it needs to be fortified to support postnatal growth. Standard fortification with a multicomponent fortifier (MCF) cannot always provide breastfed preterm infants with sufficient amounts of nutrients (1–5). Individualized fortification (adjustable or targeted) has been proposed to improve nutritional support (6, 7). Adjustable fortification relies on monitoring blood urea nitrogen. Protein is added when urea is low (8). This improves the ratio of protein-to-energy intake, which can support gains in weight and head circumference (2, 6, 9, 10). Targeted fortification relies on the analysis of HM composition followed by the addition of protein and/or energy to reach a target composition for covering the theoretical needs of the infant (3.5–4.5 g protein/kg/d and 110–135 kcal/kg/d) (11). However, targeted fortification has been shown to improve only weight gain (not length and head circumference) (2, 7), and a randomized trial failed to show a benefit in growth (12). New MCFs and a specifically designed protein fortifier (PF) powder were recently made commercially available in Europe, allowing better individualization of HM fortification.

Similar to the milk of most mammalian species, the osmolarity of unfortified HM is around 300 mOsm/l (approximately an osmolality of 338 mOsm/kg) (13–15). The presence of micro- and macro-nutrients in MCFs increases the total osmolality (14, 16, 17). Fortification has been thought to lead to an increase in osmolality because HM amylase activity induces hydrolysis of the dextrin (polysaccharides) content of fortifiers, leading to the production of small osmotically active mono- or di-saccharides (5, 13, 16). Glucose polymers are the main source of carbohydrate in most fortifiers because of their lower osmotic activity per unit weight compared to lactose or monosaccharides. High osmolality significantly alters gut mucosal integrity in animals and has been suspected to increase the risk of digestive intolerance and necrotizing enterocolitis in infants (5, 13, 14). Although the evidence is not that strong, it is often considered that the osmolality of fortified HM should remain below 450 mOsm/kg (an osmolarity of 400 mOsm/l) (13). As previous studies showed that the osmolality of HM fortified with older fortifiers increased when it is prepared 24 h before administration, it has been suggested that HM should be fortified at the patient's bedside (16, 18).

We aimed to evaluate the osmolality of HM fortified with available products, from fortification to 24 h after adding the fortifier. We assessed the impact of each fortifier on the osmolality and whether or not bedside fortification is useful for preventing osmolality increase.

## Materials and methods

### HM samples

The study involved the regional Auvergne-Rhone-Alpes Milk Bank in Lyon, France. The HM used for the study was unsuitable for use by premature babies due to significant bacteriological contamination (revealed by pre-pasteurization bacteriological testing). Donors provided written consent for use of their milk for research purposes. The HM came from three different donors because we aimed to select HM samples with a wide range of protein and energy contents. According to analysis using infrared spectroscopy (Miris® Uppsala, Sweden), protein and energy concentrations ranged from 1 to 1.6 g/dl and from 60 to 101 kcal/dl, respectively.

### Preparation of fortified HM

The preparation of food for hospitalized preterm infants is performed by dedicated staff in a dedicated room close to the regional HM bank that is in the same building as the neonatal intensive care unit at Croix Rousse University Hospital. The milk is stored in a freezer (−20°C) and thawed during preparation. To explore different types of fortifier with different compositions, we prepared samples of fortified HM using the three MCFs available in France in 2016: Suppletine®, which is produced by Lactalis (Laval, France), Fortema® (also called Aptamil® in other European countries), which is produced by Bledina (Villefranche-sur-Saône, France), and Fortipré®, which is produced by Nestlé (Marne la Vallée, France), which were designated MCF1, MCF2, and MCF3, respectively. These samples were prepared with or without the PF Nutriprem®, which is produced by Bledina (Villefranche-sur-Saône, France) (Table 1). We added a quantity of each MCF sufficient to cover the protein and energy needs that allowed an enteral intake of 160 ml/kg/day (11). This quantity amounted to 4 g of MCF1, MCF2, or MCF3 in 100 ml HM. The amount of PF added to the HM was either 0.5 g or 1 g per 100 ml HM, which were designated PF1 and PF2, respectively. The recently commercialized PF was specifically designed to increase the protein-to-energy ratio of milk ingested by poorly growing infants or to allow targeted fortification, a strategy that has been shown to be efficient for short-term growth (2, 6, 7). The amount of each fortifier added to the HM was weighed by a precision balance to the nearest 0.1 g, as done routinely by the dedicated staff.

**Table 1:** Composition of the multicomponent fortifiers (MCFs) and protein fortifier (PF) (per gram of powder).

	**Multicomponent fortifiers**			**Protein fortifier**
	**MCF1 Suppletine® (Lactalis)**	**MCF3 Fortipre® (Nestle)**	**MCF2 Fortema® (Bledina)**	**PF Nutriprem® (Bledina)**
Energy (kcal)	3.5^*^	3.5^*^	3.5^*^	3.4^*^
Protein (g)	0.23^**^	0.20^**^	0.25^**^	0.82^**^
Na (mg)	7.5	5.2	8.0	7.8
K (mg)	4.5	13.2	5.3	12.3
Ca (mg)	13	15	14.9	5.2
Ph (mg)	8.7	9.0	8.7	5.2
Iron (mg)	0	0.3	0	0

* *Energy source: carbohydrates*.

** *Partially hydrolyzed source*.

### Measurement of osmolality

Osmolality is a measure of osmolar concentration and is defined as the number of osmoles of solute per kilogram of solvent, expressed as mOsm/kg. It was blindly assessed using the freezing point technique [micro-osmometer automatic ADVANCED 3300, Radiometer S.A.S (Neuilly-Plaisance, France) France]. The freezing point of a solution is altered in direct relation to the amount of solute in solution. The reproducibility of the osmolality assessment based on 11 successive measurements in two HM samples was 0.75%.

#### First experiment: evaluation of osmolality due to fortifier addition to HM under routine conditions.

We assessed the osmolality at 2 (H2) and 24 (H24) h after the addition of each MCF with or without PF, which reflected routine practices. Indeed, as fortified HM is prepared in the dedicated room close to the HM bank, preterm infants generally receive their first meal no earlier than 2 h after its preparation. Between H2 and H24, the milk was stored at 4°C, just as it is in routine practice, prior to dispensation to premature infants. We calculated the percentage of increase in osmolality between H2 and H24.

#### Second experiment: early osmolality kinetics

To precisely investigate the early evolution of osmolality between H0 and H2, we fortified HM with MCF1 or MCF2 and measured the osmolality after fortification at 0, 1, 5, 10, 15, 20, 30, 40, 50, 60, 90, and 120 min. A sample of each preparation was stored at 4°C and the osmolality was measured 24 h later to calculate the proportion of the increase that occurred early after fortification.

### Statistics

Osmolality values were presented as mean and one standard deviation. Comparison between H2 and H24 were performed using a Wilcoxon test.

## Results

Osmolality was assessed in 30 samples of unfortified or fortified HM. The mean (SD) osmolality of unfortified HM (*n* = 3) was 293 ± 4 mOsm/kg at H2 and it did not increase significantly by H24 (295 ± 5 mOsm/kg, i.e., +0.8%).

**Figure 1:**
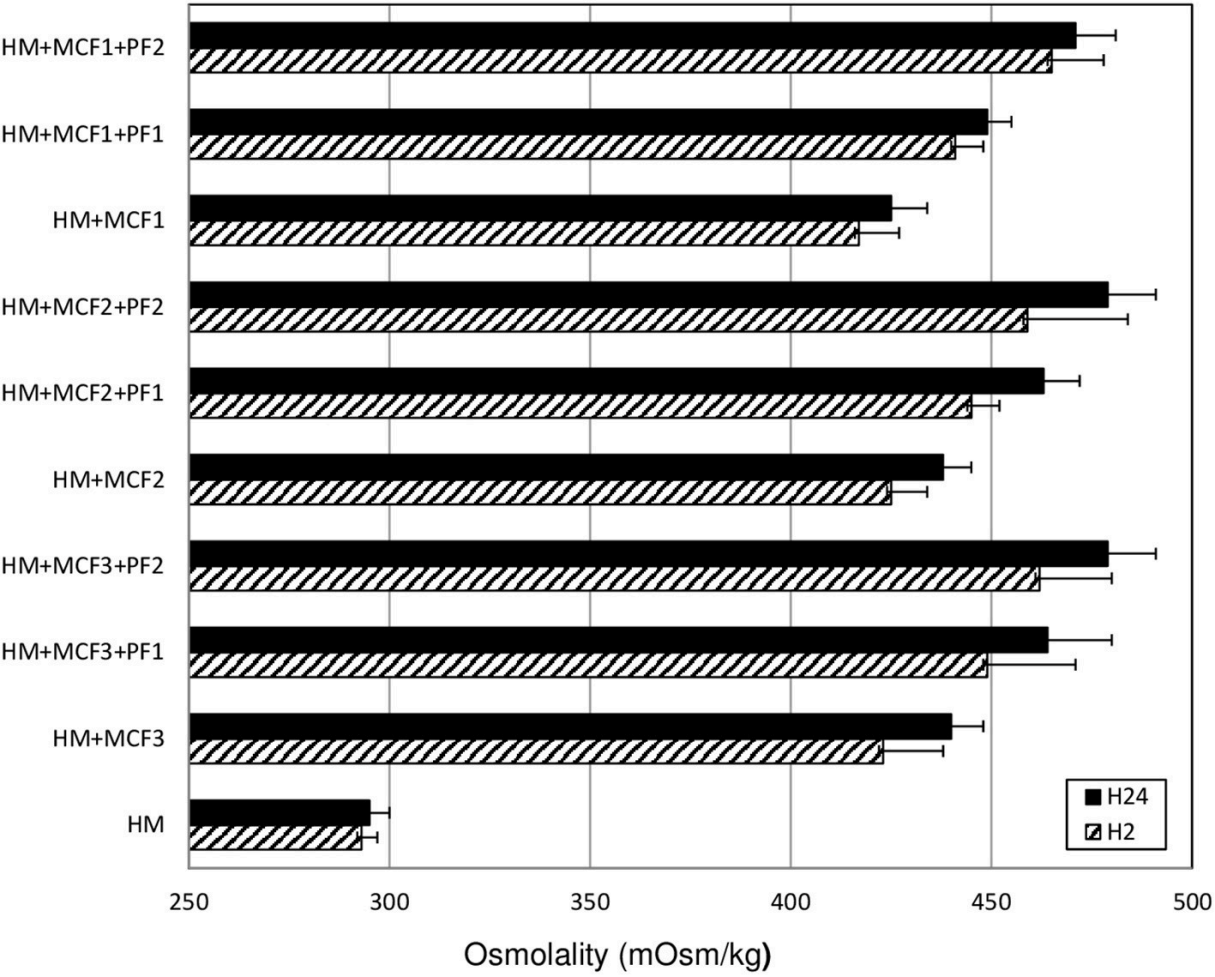
Mean osmolality of human milk assessed at 2 (H2) and at 24 (H24) hours after the addition of three different multicomponent fortifiers: MCF1 (Suppletine®, Lactalis), MCF2 (Fortema®, Bledina), or MCF3 (Fortipré®, Nestlé) at 4 g per 100 ml of human milk, with or without protein fortifier (Nutriprem®, Bledina) at 0.5 g (PF1) or 1 g (PF2) per 100 ml of human milk. HM: unfortified human milk. No significant difference between H2 and H24 (Wilcoxon test).

The mean (±SD) osmolality of fortified HM (*n* = 27) increased significantly between H2 (443 ± 21 mosm/kg) and H24 (457 ± 20 mosm/kg). The mean increase in osmolality was 3.1% (*p* < 0.01) (range: 0.2–10.8%). However, at H24, the osmolality was over 400 mOsm/kg for all samples and over 450 mOsm/kg for 17 out of the 27 samples (63%) (Figure 1).

The increase in osmolality was significant and was of similar amplitude for different types of fortification. When adding an MCF alone (*n* = 9 samples), the osmolality increased from 422 ± 11 mosm/kg at H2 to 434 ± 10 mosm/kg at H24 (*p* < 0.01). When using MCF+PF1 (*n* = 9), it increased from 443 ± 6 to 458 ± 10 mosm/kg (*p* < 0.01), and when using MCF+PF2 (*n* = 9), in increased from 462 ± 17 to 476 ± 11 mosm/kg (*p* < 0.01) (Figure 1).

**Figure 2:**
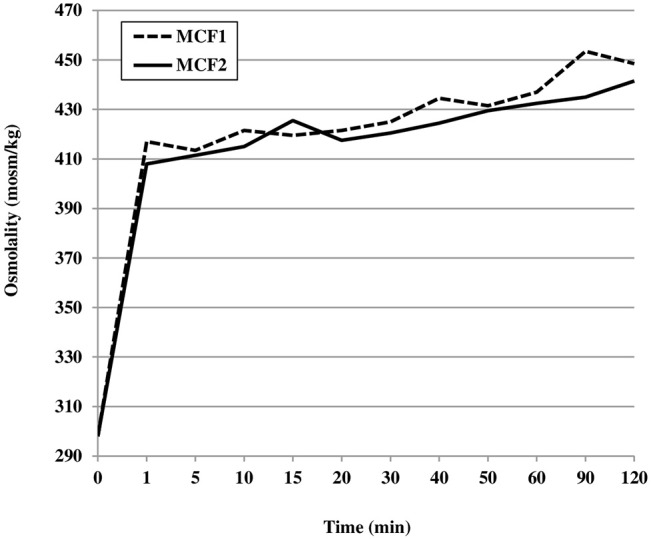
Mean osmolality of human milk assessed during the first 2 h after the addition of two different multicomponent fortifiers: MCF1 (Suppletine®, Lactalis) or MCF2 (Fortema®, Bledina).

Regarding the kinetics analysis, during the first 2 h after the addition of MCF1 or MCF2, the osmolality increased very rapidly. The increase occurred immediately (during the first minute after fortification) and was similar for both fortifiers tested: +119 mOsm/kg (+40%) for MCF1 and +110 mOsm/kg (+37%) for MCF2. This represented 79 and 76% of the total increase for MCF1 and MCF2, respectively Figure 2.

## Discussion

HM fortification induces an immediate and significant increase in osmolality. Depending on the type of fortification and amount of fortifier added to HM, the osmolality can reach values previously associated with an increased risk of digestive intolerance or necrotizing enterocolitis. A major part of that increase occurred within the first minute after addition of each fortifier.

We observed a slight increase in osmolality (+3.1%) between 2 and 24 h after the addition of MCFs. This is consistent with the previous studies, which showed that storage of HM fortified with first-generation MCFs increased osmolality by 4 and 5–10%, respectively (7, 16, 17). Such a slight increase in osmolality (+14 mosm/kg) during storage was also reported for new products available to improve the nutritional value of HM (14). Rosas et al. more recently reported a slightly greater increase in osmolality (+13–15%) (18). The increase in osmolality during storage was originally thought to be mainly attributable to the amylase in HM that breaks down the polysaccharides in the fortifier to produce molecules with higher osmolality (mono- or di-saccharides) (16, 19). The similar increase in osmolality during storage reported in the 1990s and nowadays is probably related to the similar proportion of carbohydrate (around 70/100 g) and composition (mainly or exclusively dextrin) in fortifiers.

Based on these previously reported results, Choi et al. recently wrote that osmolality was increased by 2.5–5.0% within 10 min after standard fortification, with a further 4% increase after storage at 4°C for 24 h (15). However, this key message is not fully in-line with the reality. Indeed, in all the relevant studies, the time of the initial osmolality assessment was either unreported or was at least 5–10 min after the addition of the fortifier (14, 16–18). The “baseline” osmolality values were already at very high levels. Kriessl et al. presented median “baseline” values—measured “immediately” after fortification—that were already between 475 and 691 mOsm/kg (14). These authors reported a slight but significant increase of 14 mOsm/kg after 24 h of storage at 4°C, which was considered to be of negligible clinical relevance (14). Rosas et al. presented mean “Time 0” values—actually measured at 5 min after fortification—that were between 384 and 486 mOsm/kg (18). Because the increase between the basal value and the value at 5 min after addition of the fortifier represented 59–72% of the total increase in osmolality, Rosas et al. suggested that infant feeding should occur within 5 min after the addition of the fortifier. By measuring the osmolality very early after addition of the fortifier, we were able to show that >70% of the increase occurs during the first minute. Thus, it is clear that the major part of the osmolality increase was not related to the progressive transformation of carbohydrates during the 24-h storage at 4°C prior to administration to infants (16). Instead, it was due to the instant addition of solutes (osmoles) to the HM. Therefore, the main factor explaining the osmolality increase was the amount of fortifier added, as previously suggested by Choi et al. (15). This implies that efficient prevention of increased osmolality should not rely on carrying out bedside fortification to shorten the time between fortifier addition and administration of fortified HM. Furthermore, the recommendation regarding bedside fortification could be deleterious because not all neonatal intensive care units have a dedicated room and dedicated staff to precisely weigh the fortifier and add it in safe hygienic conditions, and fortification should only be considered “safe” when the amount of fortifier can be precisely weighed and added to milk in hygienic conditions.

Notably, the osmolality values reported by previous studies were higher than our values, which could be due to the amount or type of fortifier used. The amount of fortifier tested by other researchers was sometimes greater than the amount recommended by the manufacturer, which was probably done to determine the upper limit that should not be exceeded (14, 18). However, when, in previous studies, the highest dose of protein supplement (4 g) was added to MCF-fortified HM containing 1.8 g protein/dL, it would have led to a protein intake of 7.1 g protein/kg/day for an enteral intake of 160 ml/kg/day (13, 14). Even if this type of fortification (4 g protein supplement) was added to HM containing only 1 g protein/dL, protein intake would have been 5.6 g/kg/day, which is fairly high. Furthermore, such fortification was associated with osmolality values above 600 mOsm/kg (14). These results show that it is crucial to use protein supplements very carefully. Rosas et al. also reported that very high amounts of fortifier increased osmolality up to 500–550 mOsm/kg (18). In our study, we measured HM protein and energy contents and fortified the HM with quantities of MCF and PF required to cover protein and energy needs according to current recommendations (11). This explains why we observed lower osmolality values than those reported by previous studies.

As fortifiers are considered to be food rather than health products, manufacturers are not obliged to provide clinical evaluation before launching these products. However, it should be mandatory to provide clinicians with a precise evaluation of the impact of each new product on the osmolality HM. Choi et al. recently showed that the osmolality fortified HM has a linear relationship with the quantity macronutrients added. These authors proposed a prediction model to predict the osmolality of HM fortified using targeted fortification (16). HM was fortified with North American MCFs (including lipids as an energy source) and supplementary nutrients (protein and/or lipids and/or glucose polymer). The model was validated using specific products available in Canada at that time. However, the model is product-specific and therefore not transferable to neonatal intensive care units that use different products or different fortification strategies. Therefore, evaluating the osmolality of fortified HM is necessary to ensure that safe food is prepared for preterm infants. Such an evaluation should be performed independently from the manufacturer, as Kriessl et al. reported a greater increase in osmolality compared to the values provided by the manufacturer (14).

A limitation of our study is that we did not test cow milk-based fortifiers that use lipids together with carbohydrates as an energy source or HM-based fortifiers. These cow milk-based fortifiers were available only in North America at the time of the study and HM-based fortifier is still not currently available in France. Cowmilk-based fortifiers containing lipids are interesting as they could help to reduce the osmolality of fortified HM. However, Rochow et al. reported that an MCF containing lipids enhanced the osmolality of HM from 295 to 405 mOsm/kg, and to 436 mOsm/kg after targeted fortification (12). In contrast, it has been nicely shown by Choi et al. that the addition of a fat supplement to HM minimally decreased the osmolality (15). The partial replacement of carbohydrates by fat in an MCF may help to reduce the osmotic load and thus the osmolality of fortified HM. The first cow milk-based fortifier containing lipids was evaluated by Rigo et al. (20) and became available for European users in 2017. As expected, the osmolality (390 mOsm/kg) was reduced when compared to previous cow milk-based products and the control MCF (which led to an osmolality of 441 mOsm/kg) (20). Although the study's main objective was to evaluate the effect of the cow milk-based fortifier on growth, digestive tolerance was also evaluated and was similar to that for the control MCF (20). Further investigations are needed to evaluate the osmolality effects of such products under routine conditions, and whether or not they could have an impact on digestive tolerance. Regarding HM-based fortifiers, it has been shown that they lead to a lower osmolality (391–412 mosm/kg) than cow milk-based products (431 mosm/kg) (21). In settings with a high prevalence of necrotizing enterocolitis (16%), HM-based fortifiers have a beneficial preventive effect against necrotizing enterocolitis among preterm infants (22). However, the reality of this benefit is still debated, notably when the prevalence is close to the common level (3–5%) (23).

Another study limitation is that we evaluated osmolality, focusing on the level of 450 mOsm/kg that was proposed as an upper limit by the American Academy of Pediatrics (AAP) in 1976, despite the fact that there is no strong evidence regarding the benefit of this limit (14, 24), Furthermore, there is a difference between the measured osmolality and the effective osmolality *in vivo* because not all substances create an osmotic gradient *in vivo* (25). Molecules that do not lead to an osmotic gradient across the intestinal membrane *in vivo* are not likely to increase the risk of necrotizing enterocolitis due to osmolality (25). Moreover, when particles contributing to osmolality are present in the gut lumen, the normal physiological response is the secretion of hypo-osmolar fluid to reduce the osmolality of the luminal content (13, 26). However, necrotizing enterocolitis is a multifactorial complication of prematurity, and each risk factor, such as using high-osmolality products for enteral nutrition, should be avoided. It is notable that the studies on which the AAP recommendations were based did not take important confounding variables, such as hyperosmolar therapeutic additives and oral drugs, into account (13). Furthermore, the formulas used in the studies reviewed by the AAP had an osmolality in excess of 500 mOsm/kg. A recent meta-analysis of 11 trials (882 infants) of nutrient fortification have not shown evidence of an increase in necrotizing enterocolitis associated with fortification (3). Despite this, while waiting for more precise data, the limit of 450–500 mOsm/kg could be considered appropriate. Our results contribute to improving clinicians' knowledge about the effects of HM fortification, and how to avoid increased osmolality.

In conclusion, the available fortifiers induce a significant increase in osmolality to levels usually considered to be associated with an increased risk of necrotizing enterocolitis. As there are differences in the effect of each type of fortification on HM osmolality, the choice of fortifier should be carefully analyzed. The increases in osmolality occurred immediately after the addition of fortifiers, suggesting that bedside fortification is not the key factor to be taken into account to reduce osmolality of fortified HM. Further research should focus on improving fortifier composition to cover infants' nutritional needs while also keeping osmolality as low as possible.

## Author contributions

J-CP, RB, and PP designed and directed the project, and wrote the paper with input from all authors. NK, DM-M, and VC measured the osmolality. DM-M, VC, J-CP, RB, PP, and NK analyzed the data.

### Conflict of interest statement

The authors declare that the research was conducted in the absence of any commercial or financial relationships that could be construed as a potential conflict of interest.
